# Effort-reward imbalance and common mental disorders among public sector employees of Iran: a cross-sectional analysis

**DOI:** 10.1186/s12889-024-18871-6

**Published:** 2024-05-30

**Authors:** Nastaran Nasirpour, Mohammadreza Shalbafan, Ebtesam Savari, Ahmad Pirani, Hamid Reza Baradaran, Abbas Motevalian

**Affiliations:** 1 Department of Epidemiology, School of Public Health, Iran University of Medical Sciences, Tehran, Iran; 2 Mental Health Research Center, Psychosocial Health Research Institute (PHRI), Department of Psychiatry, School of Medicine, Iran University of Medical Sciences, Tehran, Iran; 3 Endocrine Research Center, Endocrinology & Metabolism Research Institute, Iran University of Medical Sciences, Tehran, Iran; 4 Research Center for Addiction and Risky Behaviors (ReCARB), Psychosocial Health Research Institute (PHRI), Iran University of Medical Sciences, Tehran, Iran

**Keywords:** Job stress, Mental disorders, Effort-reward imbalance

## Abstract

**Background:**

The effort-reward imbalance (ERI) model is a widely used theoretical model to measure stress in the workplace. The objective of this study was to investigate the relationship between ERI and three common mental disorders: major depressive disorder (MDD), generalized anxiety disorder (GAD), and obsessive-compulsive disorder (OCD).

**Methods:**

In this cross-sectional analysis, the study sample consisted of 4453 baseline participants of the Employees’ Health Cohort Study of Iran (EHCSIR). Trained psychologists utilized the Persian version of the Composite International Diagnostic Interview (CIDI-2.1) during the baseline assessment to identify common mental disorders. Additionally, the validated Persian version of the 23-item ERI questionnaire was employed to assess effort, reward, overcommitment, and effort-reward ratio. To examine the association of ERI components with three common mental disorders (MDD, GAD, and OCD) over the past twelve months, multiple logistic regression analyses were conducted.

**Results:**

The prevalence of effort-reward imbalance in the study sample was 47.1%. Higher ERI score was significantly associated with MDD (OR: 3.43, 95% CI: 2.30–5.13), GAD (OR: 2.42, 95% CI: 1.27–4.63), and OCD (OR: 2.23, 95% CI:1.19–4.19). The study participants who reported higher scores on work overcommitment had a higher likelihood of having MDD (OR: 1.16, 95% CI:1.10–1.23), GAD (OR: 1.07, 95% CI: 1.01–1.14), and OCD (OR: 1.19, 95% CI: 1.09–1.29).

**Conclusions:**

According to the study’s findings, work-related stress, as determined by the ERI model, is a significant factor in the development of common mental disorders among employees in the public sector.

## Background

The global prevalence, of major depressive disorder (MDD) and anxiety disorders stands at 4.7% and 8.3% respectively over 12 months. However, in Iran, the prevalence of these disorders is higher with MDD affecting, around 12.7% of the population and anxiety disorder impacting 15.6% [1, 2]. Mental disorders are progressively emerging as the predominant factor necessitating sick leave and prolonged work disability [3]. Mental well-being is linked to heightened productivity, creativity, enhanced social interactions, and elevated socioeconomic standing [3].

Stressful socio-environmental conditions are a pivotal factor influencing the development of mental disorders in workplace settings [4]. The Effort-Reward Imbalance (ERI) model, developed by Siegrist, stands as a widely accepted theoretical framework for assessing workplace stress [5, 6]. This model centers on the notion of an imbalance between the efforts exerted at work and the rewards garnered in return. Furthermore, the ERI model encompasses an overcommitment element, a personality trait that amplifies work-related stress and heightens susceptibility to this imbalance [4, 7]. A systematic review of 18 studies found that the range of effort-reward imbalance was between 4 and 81% [8]. Moreover, a cross-sectional study conducted in five educational medical centers in Iran reported that the prevalence of ERI was 55% among nurses [9]. Health workers who work under intensive workloads are at risk of experiencing ERI [8, 10].

The epidemiological evidence regarding the association between workplace stress and mental health is predominantly derived from high-income countries, specifically limited to the investigation of depression [11–14]. Based on the findings of two meta-analysis studies, it has been determined that exposure to ERI is a significant risk factor for the development of stress-related disorders and mental illnesses [15, 16]. Similarly, a cross-sectional study among Chinese public health workers revealed that effort and overcommitment were positively associated with depression and anxiety, while the reward was negatively associated with these disorders [17]. However, another similar study did not confirm the relationship between ERI factors with MDD and generalized anxiety disorder (GAD) [18]. Some studies have shown that the effect size of the effort-reward ratio is greater than the effect size of individual effort or reward factors [19]. To date, there has been no research conducted on the impact of the ERI model on obsessive-compulsive disorder (OCD). However, it is recognized that stress in the work environment can contribute to the development of this disorder [20].

The possible association between the ERI model and mental disorders may be ascribed to physiological, biological, and lifestyle risk factors [21, 22]. This imbalance can result in the experience of negative emotions and sustained autonomic activation, which in turn can have detrimental long-term effects on mental health [7].

The ERI model can be utilized as a valuable instrument for recognizing work-related stress and directing workplace health promotion initiatives. Such programs hold significant importance in low-income nations, particularly during swift and stressful transformations in the labor market.

The present study is a cross-sectional analysis of the association between the ERI model (effort, reward, overcommitment, and effort-reward imbalance) and twelve-month MDD, GAD, and OCD, using the baseline data from the Employees’ Health Cohort Study of Iran (EHCSIR). We hypothesized that (1) effort, overcommitment, and ERI scores would act as risk factors, (2) reward would serve as a protective factor, and (3) the effect size of the ERI score would be stronger than the effect sizes related to the individual model components of effort or reward.

## Methods

### Study design

This is a cross-sectional study that utilizes baseline data from the Employees’ Health Cohort Study of Iran (EHCSIR). EHCSIR is a prospective cohort study that aims to investigate the various occupational and non-occupational determinants contributing to non-communicable diseases among Iranian public sector employees. The reference population for the EHCSIR comprised over 15,000 employees working across 43 distinct units within Tehran province, predominantly in schools, hospitals, health centers affiliated with the Iran University of Medical Sciences, and the headquarters staff of the Ministry of Health and Medical Education. All active non-temporary staff were eligible to participate in the cohort study. Initially, the study aimed to recruit 10,000 employees; however, due to COVID-19 social distancing restrictions, recruitment was halted in March 2020. From July 2017 to March 2020, a total of 4,886 employees were successfully recruited [23].

### Baseline assessments

All of the 4,886 participants underwent a comprehensive baseline assessment lasting approximately 7 h at a single center. This assessment encompassed several face-to-face interviews (covering medical, psychological, nutritional, and sociodemographic items), clinical examinations (including blood pressure, electrocardiogram, pulmonary function tests, and auditory and visual assessments), anthropometric measurements, and the collection of biospecimens (blood and urine) for laboratory tests. Additionally, participants completed a tablet-based self-administered questionnaire [23].

### Measurements

#### Effort-reward imbalance (ERI)

To assess the effort-reward imbalance, a trained psychologist utilized the 23-item Persian version of the ERI Questionnaire [24], in a personal interview during the baseline assessment. The Persian version of the ERI Questionnaire demonstrated satisfactory validity and reliability among employees of Iran [24]. The Cronbach’s alpha reliability coefficients for the constructs of effort, reward, and overcommitment were determined to be 0.61, 0.85, and 0.67, respectively. Furthermore, the item-total correlations for all constructs exceeded the threshold of 0.23, and the item-scale correlations were found to be higher than 0.4 [24]. Each item had a four-item Likert scale response including “completely agree = 1”, “agree = 2”, “disagree = 3”, and “completely disagree = 4”. The “effort”, “reward”, and “overcommitment” scores were calculated based on the average scores of the relevant 5, 11, and 6 items, respectively. The “ERI” score was calculated by dividing the effort score by to reward score.

#### Diagnosis of common mental disorders

To evaluate various psychiatric disorders, including major depressive disorder, dysthymia, obsessive-compulsive disorder, and generalized anxiety disorder, the Lifetime Version of the Composite International Diagnostic Interview (CIDI, version 2.1) was utilized. The CIDI is a fully structured psychiatric diagnostic interview developed by the World Health Organization which assesses psychiatric disorders based on DSM and ICD criteria. Trained interviewers who can be individuals without clinical experience manage to accurately utilize this diagnostic tool [2, 25]. Extensive testing across various languages and settings has revealed its strong reliability and validity in the majority of its diagnostic categories [2, 26, 27]. The Persian version of CIDI 2.1 has been found to have acceptable validity and reliability [28, 29]. Moreover, the inter-rater reliability of the CIDI 2.1 was evaluated within a general population sample, yielding kappa coefficients of 0.5 or higher for various psychiatric disorders [29]. It is capable of categorizing depressive disorders based on type (single or recurrent) and severity (mild, moderate, and severe) [30].

During the baseline assessments of the EHCSIR study, trained psychologists conducted interviews and assessed common mental disorders using the Persian lifetime version of the Composite International Diagnostic Interview (CIDI-2.1) [29], based on DSM-IV criteria. The assessment included Major Depressive Disorder (MDD), which encompassed all mild, moderate, and severe cases of both single and recurrent episode major depressive disorders. The most recent episode among subjects who had ever met the full criteria for the disorder in their lifetime was used to recognize the twelve-month disorder.

#### Covariate factors

The EHCSIR baseline dataset was used to extract socio-demographic factors, which included gender, age, marital status, education, wealth index, job, and workplace. Furthermore, Job strain, defined as high levels of psychological demand with low control, was measured using the Persian version of the Job Content Questionnaire (JCQ) [31]. The JCQ, developed by Karasek’s demand-control model, aimed at evaluating the psychosocial aspects of the work environment [32]. This questionnaire plays a crucial role in predicting risks associated with stress [32]. To evaluate the job strain, a trained psychologist employed the Persian version of the JCQ in a structured interview during the baseline assessment. The Persian version of the JCQ has exhibited strong validity and reliability in assessing workplace stress levels among healthcare professionals in Iran [31]. Notably, the internal consistency coefficient, as measured by Cronbach’s α, surpassed 0.75 for the majority of the questionnaire’s subscales [31].

### Ethical statement

The research ethics committee of the Iran University of Medical Science approved the study, with ethical code #IR.IUMS.REC.1402.593. All participants provided written informed consent before joining the study. Moreover, the participants’ data were kept confidential and only accessible to the main study investigators. Anonymized data was used for statistical analyses. The study was conducted by the Declaration of Helsinki [33], national guidelines, and regulations.

### Approach to missing data

The total number of EHCSIR participants was 4886 public sector employees. Among these participants, 217 did not complete the CIDI interview or did not respond to the ERI questionnaire. Specifically, 146 did not respond to the CIDI interview, 145 did not respond to the ERI, and 74 did not respond to both. Additionally, for 216 participants, some of the covariate data were missing.

Considering that non-response in most cases was due to the long time spent conducting the basic evaluations and not participating in the psychological interviews station, these cases were considered as missing completely at random (MCAR). Therefore, a complete data analysis approach was applied.

### Statistical analysis

Descriptive statistics were presented as the mean ± standard deviation (SD) for continuous variables and as numbers (percentage) for categorical variables. We utilized principal component analysis (PCA) to estimate wealth levels based on asset indices. Job strain was considered binary as job strain (high demands and low control) versus no strain (all other categories combined).

Logistic regression analyses were conducted to investigate the association between each of the ERI model components (effort, reward, overcommitment, and ERI) and each of the common mental disorders (MDD, GAD, and OCD). We calculated the odds ratio (OR) of outcomes per 1 standard deviation increase in each component of the ERI model. Three models were used in each logistic regression analysis: no adjustment, minimal adjustment (adjusted for gender, age, marital status, education, and wealth index), and full adjustment (adjusted for gender, age, marital status, education, wealth index, job, workplace, and Job strain model). Adjusted odds ratios and their corresponding 95% confidence intervals (CIs) were presented. STATA version 14 was used for all analyses, and the significance level was set at 0.05.

## Results

In the current study, out of the total 4886 participants enrolled in the EHCSIR, 4453 individuals had complete data and were included for analyses. 47.1% (*n* = 2097) of respondents had effort-reward imbalance. This proportion was 50.1% (821 out of 1640) among men, and 45.3% (1274 out of 2813) among women. The study population characteristics are presented in Table 1. The average age of the participants was 42.9 years (SD = 8.1), and the majority of them were women (63.2%). Additionally, 80.8% of the subjects were married.

Mean and standard deviation for each factor of the ERI model are given in Table 2. Men showed higher levels of effort (< 0.01), and job promotion (< 0.05), than women. However, women rated their job security much higher than men (< 0.001). Moreover, the mean effort-reward ratio was 1.02 (SD = 0.26), and didn’t show a statistically significant gender difference.

Tables 3 and 4, and 5 present the associations between scores of ERI model factors and twelve-month MDD, GAD, and OCD. We found that effort did not significantly influence these mental disorders in any of the three models. Moreover, it was observed that an increase of one standard deviation in work reward had a small but significant effect on reducing the likelihood of having mental illnesses.

In Table 3, fully adjusted Model 3 indicates that a one standard deviation increase in the overcommitment score revealed a weak yet statistically significant association with MDD, yielding an OR of 1.16 (95% CI: 1.10–1.23). Additionally, a higher ERI score by one standard deviation demonstrated a strong positive correlation with MDD, as reflected by an odds ratio of 3.43 (95% CI: 2.30–5.13). Similarly, Table 4 shows that a one standard deviation increase in the overcommitment score displayed a weak OR for GAD, measuring at 1.07 (95% CI: 1.01–1.14). Moreover, the odds of GAD were found to be 2.42 times higher for each standard deviation increase in the ERI score (95% CI: 1.27–4.63). As demonstrated in Table 5, the overcommitment score was associated with OCD (OR: 1.19, 95% CI: 1.09–1.29). Furthermore, the likelihood of OCD increased by an OR of 2.23 for every standard deviation rise in the ERI score (95% CI: 1.19–4.19).

The odds ratios associated with each mental disorder in relation to the ERI score exhibited greater effect sizes compared to the ORs linked to the separate components of effort or reward within each model.

Based on our findings, none of the proposed hypotheses was rejected. However, the hypothesis suggesting that effort is a contributing risk factor for mental disorders did not receive support.

**Table 1:** Characteristics of study subjects according to twelve-month MDD, GAD, and OCD in EHCSIR

		N	MDD			GAD			OCD		
			n	%	P-value	n	%	P-value	n	%	P-value
Gender	Male	1640	75	4.6	< 0.001*	41	2.6	0.77	36	2.2	0.15
	Female	2813	219	7.8		74	2.7		82	2.9	
Age	34 ≥	716	47	6.6	0.63	18	2.6	0.39	28	3.9	0.053
	35–44	1942	138	7.1		47	2.5		43	2.2	
	45–54	1423	85	6.0		44	3.2		41	2.9	
	≥ 55	372	24	6.5		6	1.7		6	1.6	
Marital status	Married	3600	209	5.8	< 0.001*	88	2.5	0.49	92	2.6	0.25
	Never married	596	50	8.4		19	3.2		15	2.5	
	Ex-married	257	35	13.6		8	3.2		11	4.3	
Education	Primary-High school	429	18	4.2	0.09	7	1.7	0.48	8	1.9	< 0.05*
	diploma	890	68	7.6		28	3.2		30	3.4	
	Associate	425	36	8.5		14	3.4		18	4.2	
	Bachelor’s level	1661	110	6.6		43	2.6		45	2.7	
	Master’s level	825	46	5.6		17	2.1		13	1.6	
	Doctoral level	223	16	7.2		6	2.7		4	1.8	
Job	Office work	1449	110	7.6	0.31	49	3.5	0.11	45	3.1	0.22
	Academic	133	7	5.3		2	1.5		2	1.5	
	Health Professional	2017	125	6.2		44	2.2		44	2.2	
	Service work	854	52	6.1		20	2.4		27	3.2	
Workplace	Educational\office	1728	112	6.5	0.50	46	2.7	0.23	43	2.5	0.78
	Public health centers	774	45	5.8		26	3.4		23	3	
	Hospitals	1951	137	7.0		43	2.3		52	2.7	
Wealth index	Low	1155	74	6.4	0.62	25	2.2	0.19	32	2.8	0.16
	Medium	2261	157	6.9		68	3.1		67	3.0	
	High	1037	63	6.1		22	2.2		19	1.8	
Job strain	No	3837	225	5.9	< 0.001*	85	2.3	< 0.001*	96	2.5	0.13
	High demands and low control	616	69	11.2		30	5.0		22	3.6	

^*^Significant

**Table 2:** Means and SDs for ERI questionnaire scales by gender

	Total		Men		Women		*P*-value
	Mean	SD	Mean	SD	Mean	SD	
Effort	2.68	0.45	2.70	0.43	2.66	0.47	< 0.01*
Reward	2.70	0.36	2.69	0.35	2.70	0.37	0.86
Esteem	2.83	0.43	2.84	0.40	2.82	0.44	0.06
Job promotion	2.57	0.44	2.59	0.43	2.56	0.44	< 0.05*
Job security	2.64	0.55	2.55	0.53	2.69	0.55	< 0.001*
Overcommitment	2.54	0.34	2.55	0.33	2.54	0.34	0.64
Effort-reward ratio	1.02	0.26	1.02	0.25	1.01	0.27	0.17

*Significant

**Table 3:** Logistic regression analysis of factors in the ERI model on twelve-month MDD

MDD	Model 1		Model 2		Model 3	
	OR	95% CI	OR	95% CI	OR	95% CI
Effort	1.03	0.99–1.08	1.03	0.98–1.07	1.03	0.98–1.07
Reward	0.88	0.85–0.90*	0.88	0.85–0.90*	0.88	0.86–0.91*
Overcommitment	1.15	1.09–1.21*	1.16	1.10–1.23*	1.16	1.10–1.23*
ERI	3.68	2.53–5.34*	3.65	2.49–5.37*	3.43	2.30–5.13*

Model 1 was unadjusted؛ Model 2 was adjusted for gender, age, marital status, education, and wealth index؛ Model 3 was also adjusted for job, workplace, and Job strain model

^*^Sig. *p* < 0.001

**Table 4:** Logistic regression analysis of factors in the ERI model on twelve-month GAD

GAD	Model 1		Model 2		Model 3	
	OR	%95 CI	OR	95% CI	OR	95% CI
Effort	1.00	0.94–1.07	1.00	0.93–1.07	1.01	0.93–1.08
Reward	0.90	0.86-0.94^c^	0.89	0.86-0.94^c^	0.91	0.87-0.95^c^
Overcommitment	1.07	1.01-1.14^a^	1.07	1.01-1.14^a^	1.07	1.01-1.14^a^
ERI	2.49	1.37-4.50^b^	2.56	1.39-4.73^b^	2.42	1.27-4.63^b^

Model 1 was unadjusted؛ Model 2 was adjusted for gender, age, marital status, education, and wealth index؛ Model 3 was also adjusted for job, workplace, and Job strain model

^a^Sig. *p* < 0.05

^b^Sig. *p* < 0.01

^c^Sig. *p* < 0.001

**Table 5:** Logistic regression analysis of factors in the ERI model on twelve-month OCD

OCD	Model 1		Model 2		Model 3	
	OR	95% CI	OR	95% CI	OR	95% CI
Effort	1.04	0.98–1.12	1.02	0.96–1.10	1.04	0.97–1.12
Reward	0.93	0.89-0.97^b^	0.94	0.90-0.98^b^	0.94	0.90-0.98^b^
Overcommitment	1.19	1.09-1.29^c^	1.19	1.09-1.29^c^	1.19	1.09-1.29^c^
ERI	2.41	1.34-4.33^b^	2.10	1.13-3.88^a^	2.23	1.19-4.19^a^

Model 1 was unadjusted؛ Model 2 was adjusted for gender, age, marital status, education, and wealth index؛ Model 3 was also adjusted for job, workplace, and Job strain model

^a^Sig. *p* < 0.05

^b^Sig. *p* < 0.01

^c^Sig. *p* < 0.001

## Discussion

The current study investigated the prevalence of ERI among the study population, which was found to be 47.1%. The study found that all factors of the ERI model, except effort, had a significant relationship with twelve-month MDD, GAD, and OCD in the EHCSIR. High reward was identified as a protective factor, while high overcommitment and ERI-score were identified as risk factors for each of these mental disorders. Furthermore, the study revealed that an increase in score had a significantly greater impact on MDD, GAD, and OCD compared to the individual components of high effort or low reward.

A systematic review of 18 studies on the ERI model among health workers found that the range of ERI varied from 4 to 81%. Moreover, at least 20% of the health workers faced an ERI in half of the studies. This proportion has been reported in Greece, Vietnam, Switzerland, and France as 81%, 32%, 18%, and 10%, respectively [8]. In our study, similar to lower-middle-income countries, we found a high prevalence of it. This can be attributed to several factors, such as heavy workload, especially at higher levels of health care services [34], and getting less reward for their workload [35].

The cross-sectional study we conducted offers additional evidence that the ERI model in the workplace is linked to a higher risk of mental disorders. This finding is in line with previously reported research results. According to a meta-analysis of eight cohort studies involving 84,963 employees, there is a positive association between the ERI experienced at work and an elevated risk of depressive disorders, with a pooled random-effects estimate of 1.49 (95% CI: 1.23–1.80). However, the authors acknowledge that the results of the meta-analysis may not be highly dependable due to the limited number of studies that were incorporated [12]. Similarly, in a study from the ELSA-Brazil project, which included 10,034 public sector employees, an increase in the prevalence ratio (PR) of depressive episodes was reported based on ERI factors. They reported relationships between effort (PR = 1.85), overcommitment (PR = 3.62), reward (PR = 3.44), and the effort-reward imbalance (PR = 2.47) with depressive episodes [11]. In addition, a large cohort study in Belgium found that cumulative experience and recent onset of ERI (OR for men = 2.8, OR for women = 4.6) and overcommitment (OR for men = 2.4) are associated with an elevated risk of depression [19]. Likewise, this cohort study reported associations between anxiety with ERI (OR for men = 2.3, OR for women = 4.5) and overcommitment (OR for men = 2.5) [19]. Besides, the results of a cross-sectional study in China showed relationships between effort (OR = 1.33), overcommitment (OR = 1.19), and reward (OR = 0.91) with GAD [17].

However, a cross-sectional study in the UK did not confirm the relationship between ERI factors and depression or anxiety symptoms, possibly due to the small sample size [18].

The connection between the constituents of the model and mental illnesses can be clarified by psychological mechanisms such as humiliation, helplessness, and diminished self-esteem [12], as well as physiological alterations such as the dysregulation of the hypothalamic-pituitary-adrenal axis and immune system [21]. Stress can trigger the dysregulation of neurotransmitter and hormone release, which can result in a range of symptoms associated with biological, affective, cognitive, and behavioral functions [4]. Likewise, people who overcommit, exert effort beyond normal levels, or often find themselves exposed to high demands at work may experience reduced potential to recover from job demands and increased sensitivity to disappointment when expected rewards are not met. These factors may ultimately lead to poor mental health [36].

The current investigation provides evidence in favor of the notion that the composite evaluation of effort and reward has a more potent impact on mental illnesses than individual evaluations of effort or reward. This finding is in line with the previous research [37]. It could be attributed to a combination of factors, including a synergistic effect, psychological mechanisms, and measurement validity [38]. Although, a different study that tested the same hypothesis discovered that the stressful effects of reward were more pronounced than the stressful effects of the ERI [11].

This study has several strengths. First, the study employed a valid diagnostic tool to measure MDD, GAD, and OCD, which is a notable strength [12]. This is in contrast to many previous studies that relied on hospital records, self-reporting, or symptom scales, which are more prone to reporting and recall biases. Second, this study assesses multiple mental outcomes, unlike other similar studies that have limited their analysis scope to one dimension of mental health. Previous studies on the relationship between the ERI model and mental disorders have primarily focused on depressive disorders, and data collection relied on self-reporting [12, 39]. In addition to depression, the present study has addressed GAD and OCD, which are relatively common mental disorders with a high disease burden. To the best of our knowledge, this is the first study to examine the relationship between the ERI model and OCD. Third, in contrast to the majority of prior investigations, we controlled for the job strain model (Demand-Control) while analyzing the correlation between the ERI model and each of the psychological disorders being studied. This was done because a systematic review has indicated a robust link between the high job strain model and common mental disorders [16]. Fourth, the study’s participants come from a variety of age groups, genders, and educational backgrounds, indicating a diverse sample. This diversity in the sample is likely to increase the generalizability of the study’s findings to the broader population. Fifth, the relationship between the ERI model and mental health is predominantly studied in high-income countries [11], while workers in low-income countries are more susceptible to experiencing adverse effects on their mental health. This is a matter of concern as approximately 75% of the global labor force is located in low-income countries [12].

The study has some limitations. Firstly, the utilization of a cross-sectional study design may diminish the robustness of conclusions regarding the association between variables. However, meta-analysis and review studies that are based on similar prospective studies can offer conclusions regarding predictive power, but not causation [40]. Furthermore, a study that employed Swedish national panel survey data discovered no indication of reverse causation from depression to psychosocial work stress [41]. Secondly, the generalizability of the study’s findings to the entire working population of Iran is limited since the sample only consists of individuals employed in the public sector.

## Conclusions

According to the results of our research, stressful work, as measured by the ERI model, is significantly associated with the psychological outcomes examined in the relatively homogeneous population of the EHCSIR project. The ERI demonstrated a stronger association with these outcomes compared to other factors in the model. These findings have important implications for promoting mental health in workplaces, particularly in low-income countries that are undergoing rapid and stressful changes in the labor market. To reduce the ERI and work stress, several solutions can be implemented in the workplace: independence and greater decision-making by employees on their work, reducing workload through hiring additional employees, telecommuting or flexible working hours, and providing educational opportunities. However, further research is necessary to investigate the underlying mechanisms that contribute to this association and to identify effective interventions that can help alleviate the negative effects of the ERI model on mental health.

## Data Availability

The datasets produced and analyzed during the current study are available upon request sent to the corresponding author.

## Abbreviations

ERI: Effort-Reward Imbalance
MDD: Major Depressive Disorder
GAD: Generalized Anxiety Disorder
OCD: Obsessive-Compulsive Disorder
EHCSIR: Employees’ Health Cohort Study of Iran
CIDI: Composite International Diagnostic Interview
IranMHS: Iran Mental Health Survey
JCQ: Job Content Questionnaire
MCAR: Missing Completely at Random
SD: standard deviation
PCA: Principal Component Analysis
OR: Odds Ratio
CI: Confidence Interval
PR: prevalence ratio
